# Microwave therapy for the treatment of plantar warts

**DOI:** 10.1186/s13047-023-00638-8

**Published:** 2023-06-15

**Authors:** Wendy Hagon, Jonathan Hagon, Greer Noble, Angela Brenton-Rule, Sarah Stewart, Ivan Bristow

**Affiliations:** 1 Shore Footed Podiatry, Milford, Auckland, New Zealand; 2 School of Clinical Sciences, Faculty of Health and Environmental Sciences, Auckland University of Technology, 90 Akoranga Drive, Northcote, Auckland, 0627 New Zealand; 3 Private Practice, Lymington, Hampshire, United Kingdom

**Keywords:** Warts, Human Papilloma Viruses, Foot, Electromagnetic Therapies

## Abstract

**Background:**

Plantar warts, or verrucae plantaris, are common lesions causing considerable pain during weightbearing activity. Although current treatment modalities have low success rates, microwave therapy has been introduced as a promising intervention. This study aimed to determine the effectiveness of microwave therapy for the treatment of plantar warts and to determine the clinical factors associated with plantar wart resolution.

**Methods:**

A retrospective analysis of 150 plantar warts from 45 patients treated with microwave therapy was undertaken. Binomial regression was conducted to explore clinical characteristics (age, gender, immunosuppression, impaired healing, multiple vs single wart, location of lesion, lesion diameter) associated with lesion resolution.

**Results:**

Of the total 150 plantar warts treated with microwave therapy, 125 (83.3%) warts resolved and 25 (17%) warts did not resolve. The mean (SD) total treatment sessions for resolved lesions was 2.8 (1.0). Decreasing age (*P* = 0.046) was the only clinical characteristic associated with resolution.

**Conclusions:**

This retrospective study has shown that plantar warts may be resolved with two to three sessions of microwave therapy, which may be more successful in younger populations.

## Background

Plantar warts, or verrucae plantaris, result from infection of epithelial keratinocytes by the human papilloma virus (HPV). Approximately 40% of the population is infected with HPV with a 14% annual incidence of plantar warts [1]. The highest occurrence is seen in children and adolescents [2]. Although spontaneous resolution is common in the majority of patients with intact cellular immunity [3], plantar warts can be associated with considerable pain during weightbearing, and cosmetic-related stress and embarrassment.

In some cases, plantar warts persist despite multiple treatments [4, 5] and in rare cases, these recalcitrant warts may be associated with the development of squamous cell carcinomas [6]. Few controlled studies have investigated treatment options for recalcitrant plantar warts, with many associated with adverse effects [7–10]. Over the past decades, the treatment of plantar warts has largely remained unchanged, with practitioners primarily using salicylic acid, liquid nitrogen (cryotherapy), and high-energy laser therapy [3]. More recently, microwave therapy has been introduced as a promising intervention [11]. Microwaves are a form of electromagnetic non-ionising radiation between 300 MHz-300 GHz. The application of microwaves to skin increases tissue temperature to a hyperthermic range (41°-44°), which renders tissue less capable of dissipating heat and leads to damage and apoptosis of keratinocytes [12]. Unlike cold treatment such as liquid nitrogen, heating of HPV-infected tissue has also been shown to promote induction of adaptive immunity [13–17].

Several clinical advantages of microwave therapy have been reported. Microwaves travel in straight lines which allows targeted treatment of HPV-infected tissue [18]. The therapy induces minimal, short-lived pain with no scarring. As it does not break skin at the site of application patients do not require dressings and can resume normal activity post treatment [18]. Finally, unlike ablative laser therapies, no vapour or smoke is produced, which eliminates the risk of spreading air-borne viral particles [19].

A pilot cohort study undertaken in the UK involving 32 adults with 54 refractory plantar warts demonstrated high resolution rates (75.9%) using microwave therapy [18]. However, the factors contributing to treatment success are unknown. The objective of this study was to determine the effectiveness of microwave therapy for the treatment of plantar warts and clinical factors associated with treatment success.

## Methods

### Design

Single-centre non-randomised experimental retrospective study.

### Participants

A retrospective cohort study was undertaken using medical records from patients who received microwave therapy between July 2018 and March 2022 at a single podiatry clinic in Milford, Auckland (New Zealand). At present, only a handful of microwave therapy units are being used throughout New Zealand, with the Milford clinic being the first to offer it to patients in July 2018. All patients who received microwave therapy from this date were invited to share their clinical treatment records for the purposes of this study. An a-priori sample size calculation was not undertaken, and sample size was informed by the number of patients who had been offered microwave therapy in this timeframe and consented to the study. Patients were included if they had one or more plantar wart(s) diagnosed by an experienced podiatrist and underwent microwave therapy treatment. Ethical approval was obtained from the Auckland University of Technology Ethics Committee on 27^th^ July 2021 (AUTEC 21/250). Data were only included from patients who provided written informed consent/assent for their clinical treatment records to be used for this study.

### Microwave therapy

Patients were excluded from receiving microwave therapy at the podiatry clinic if they were pregnant or breast-feeding, had metal implants in their feet or ankles, or were < 5 years of age. During the initial appointment a podiatrist recorded information about the location(s) and duration(s) of the plantar wart(s), and details about previous treatment(s).

**Fig. 1:**
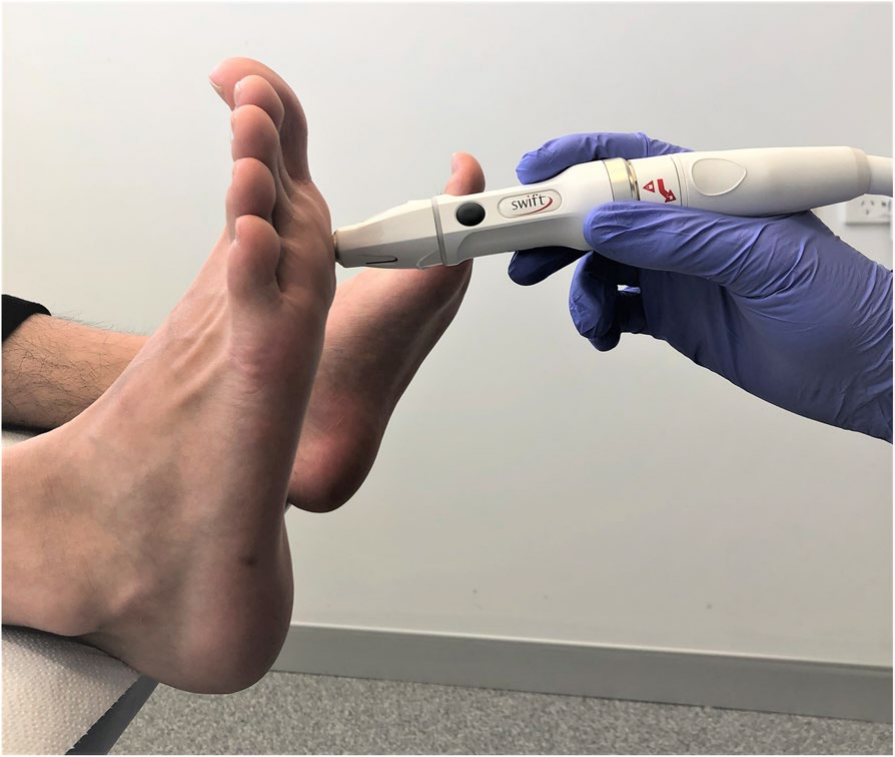
Application of the microwave therapy applicator during a treatment session

Microwave therapy was delivered using the Swift® (Emblation Ltd., UK) device, which is a hand-held tool that produces microwave heat energy within the 8 GHz range. Podiatrists at the clinic completed the manufactures standard training in using microwave therapy. The microwave energy was delivered directly to the infected tissue through a single use applicator tip to reduce the risk of cross-infection (Fig. 1). A standardised treatment procedure was followed for all patients. The first microwave therapy application at the first treatment session delivered 10 J using one application of 5W for 2 s. The wattage, frequency, and number of subsequent applications is determined by the level of pain tolerated by each patient. A 10-point scale was used at each appointment to collect patient-reported pain on arrival, during treatment, and on departure. Patients received a standard of three treatment sessions four weeks apart unless the lesion(s) resolved prior. A final review was undertaken at 12 weeks after the final treatment session where response to treatment was assessed by a podiatrist and recorded as either “resolved” or “unresolved”. Resolved lesions were defined as those no longer visible and where natural dermatoglyphics were restored. Before and after photographic evidence was used to support assessment. If unresolved at the 12-week review, patients were given the option continue further treatment sessions at 4 weeks apart until resolved, or to discontinue treatment.

### Data collection

Data from clinical treatment records of all included patients were extracted into a standardised extraction form, including patient demographics, relevant medical history, and microwave therapy and treatment parameters.

### Data analysis

The distribution of all continuous outcomes (lesion duration, total treatment sessions, average applications per session, average watts per application, average frequency setting per application, average pain, and average joules per treatment session) for both resolved and unresolved lesions were reviewed for normality using both visual inspection of histograms and formal tests of normality for skewness and kurtosis. Following confirmation of approximate normal distributions, clinical and treatment characteristics for participants were described as mean (SD) for continuous data and n (%) for categorical data. To determine clinical characteristics (age, gender, immunosuppression, impaired healing, multiple vs single wart, location of lesion, lesion diameter) that were associated with plantar wart resolution, a generalised linear model with binomial distribution and a logit link function was used. This approach was chosen as it also allowed for the inclusion of a participant-specific random effect which accounted for repeated measures taken from participants who had multiple lesions [20]. Person-specific factors (including age, sex, immune status, etc.,) result in a high-level of within-person dependence between lesions, meaning that data from different lesions within the same person will be highly correlated. The Nakagawa conditional R^2^ was computed which incorporates both fixed and random effects [21]. Adjusted estimates and *p*-values were reported. No multicollinearity was present between the clinical characteristics. All statistical analyses were performed in R (v4.1) using the lme4 package.

## Results

### Participants

A total of 135 eligible patients who received microwave therapy between July 2018 and March 2022 were invited to share their clinical treatment records for this research. A total of 47 (35%) patients responded to the invitation, including 2 who declined to participate and 45 who consented to participate. The characteristics for the 45 included patients are shown in Table 1. The majority of participants were female (*n* = 25, 56%) with a mean age of 46.6 years (range 6 to 74 years). Most participants had multiple plantar warts and had tried previous treatments which were unsuccessful including salicylic acid, cryotherapy, and cryotherapy.

**Table 1:** Participant characteristics

N		45
Gender, n (%)	Female	25 (56%)
	Male	20 (44%)
Age, mean (SD), years		46.6 (19.2)
Medical history	Dyslipidaemia	2 (4%)
	Diabetes Mellitus	1 (2%)
	Sarcoidosis	1 (2%)
	Obesity	1 (2%)
	Crohn’s disease	1 (2%)
	Asthma medication (steroid)	3 (7%)
Number of plantar warts per participant	Single	12 (27%)
	Multiple	33 (73%)
	Mean (SD)	3.3 (2.6)
	Median (range)	3.0 (1–10)
Previous treatment, n (%)	Yes	33 (73%)
	No	12 (27%)
Previous treatments^a^, n (%)	Cryotherapy	14 (31%)
	Salicylic acid	19 (42%)
	AgNO3	10 (22%)
	Needling	5 (11%)
	Surgery	2 (4%)
	Natural remedies	3 (6%)
	Pharmacy	16 (36%)
	General practitioner	8 (18%)
	Podiatrist	11 (24%)

^a^Some participants had tried more than one previous treatment

### Plantar warts

A total of 150 plantar warts were treated with microwave therapy across the 45 included patients (mean (SD) 3.3 (2.6) plantar warts per patient). Lesion-level characteristics and microwave therapy treatment details are summarised in Table 2. The majority of lesions were located on the plantar heel (*n* = 38, 25%) followed by the first metatarsal head, fifth metatarsal head, and hallux. The average lesion duration prior to treatment with microwave therapy was 35.2 months. Unresolved lesions received more treatment sessions (*P* = 0.043), a greater average number of microwave therapy applications per treatment session (*P* = 0.023), and higher average joules per treatment session (*P* = 0.034) compared to those with resolved lesions. However, average pain during treatment was significantly higher (*P* = 0.009).

**Table 2:** Lesion-level and microwave therapy treatment characteristics. Data is presented as n (%) for categorical data and mean (SD) for continuous data

			All (*n* = 150)	Resolved (*n* = 125)	Not resolved (*n *= 25)	*P*-value^d^
Location, n (%)	Rearfoot (*n* = 38)	Heel	38 (25%)	32 (26%)	6 (24%)	0.587
	Forefoot (*n* = 103)	First metatarsal head	17 (11%)	14 (11%)	3 (12%)	
		Fifth metatarsal head	17 (11%)	14 (11%)	3 (12%)	
		Hallux	16 (11%)	14 (11%)	2 (8%)	
		Second metatarsal head	15 (10%)	12 (10%)	3 (12%)	
		Fourth metatarsal head	14 (9%)	10 (10%)	4 (4%)	
		Third metatarsal head	10 (7%)	7 (7%)	3 (12%)	
		Second toe	9 (6%)	9 (7%)	0 (0%)	
		Fifth toe	3 (2%)	3 (2%)	0 (0%)	
		Third toe	2 (1%)	1 (1%)	1 (4%)	
	Midfoot (*n* = 9)	Lateral arch	5 (3%)	5 (4%)	0 (0%)	
		Medial arch	4 (3%)	4 (3%)	0 (0%)	
Foot side, n (%)		Left	68 (45%)	57 (46%)	11 (44%)	0.857
		Right	82 (55%)	67 (54%)	14 (56%)	
Duration, months, mean (SD)^a^			35.2 (41.0)	33.8 (42.2)	42.3 (35.8)	0.413
Lesion diameter, mm, mean (SD)			6.3 (7.9)	6.67 (8.6)	4.32 (1.9)	0.205
Total treatment sessions, mean (SD), range			2.8 (1.0), 1–6	2.8 (1.0), 1–6	3.2 (1.0), 1–5	**0.043**
Average applications per treatment session, mean (SD)			2.0 (0.8)	1.9 (0.8)	2.3 (0.7)	**0.023**
Average watts setting per application, mean (SD)			7.5 (2.2)	7.4 (2.1)	7.9 (2.3)	0.335
Average frequency setting per application, mean (SD)			6.2 (2.8)	6.4 (3.0)	5.6 (1.7)	0.213
Average pain^b,c^ on arrival per treatment session, mean (SD)			0.71 (1.2)	0.8 (1.2)	0.0 (0.0)	** < 0.001**
Average pain^b,c^ during treatment per treatment session, mean (SD)			7.8 (1.1)	7.7 (1.1)	8.4 (0.9)	**0.009**
Average pain^b,c^ on departure per treatment session, mean (SD)			1.1 (1.5)	1.2 (1.5)	0.7 (1.3)	0.288
Average joules per treatment session, mean (SD)			155.9 (68.3)	150.6 (54.3)	182.3 (82.4)	**0.034**

^a^Data available from only 13 (29%) of participants

^b^Data was available from 35 (78%) of participants

^c^As determined by a 0 to 10 Likert scale ranging from 0 (no pain) to 10 (extreme pain); ^d^Difference between resolved and unresolved lesions. Chi-square tests were used for categorical data, and independent t-tests for continuous data

### Effectiveness of microwave therapy

**Fig. 2:**
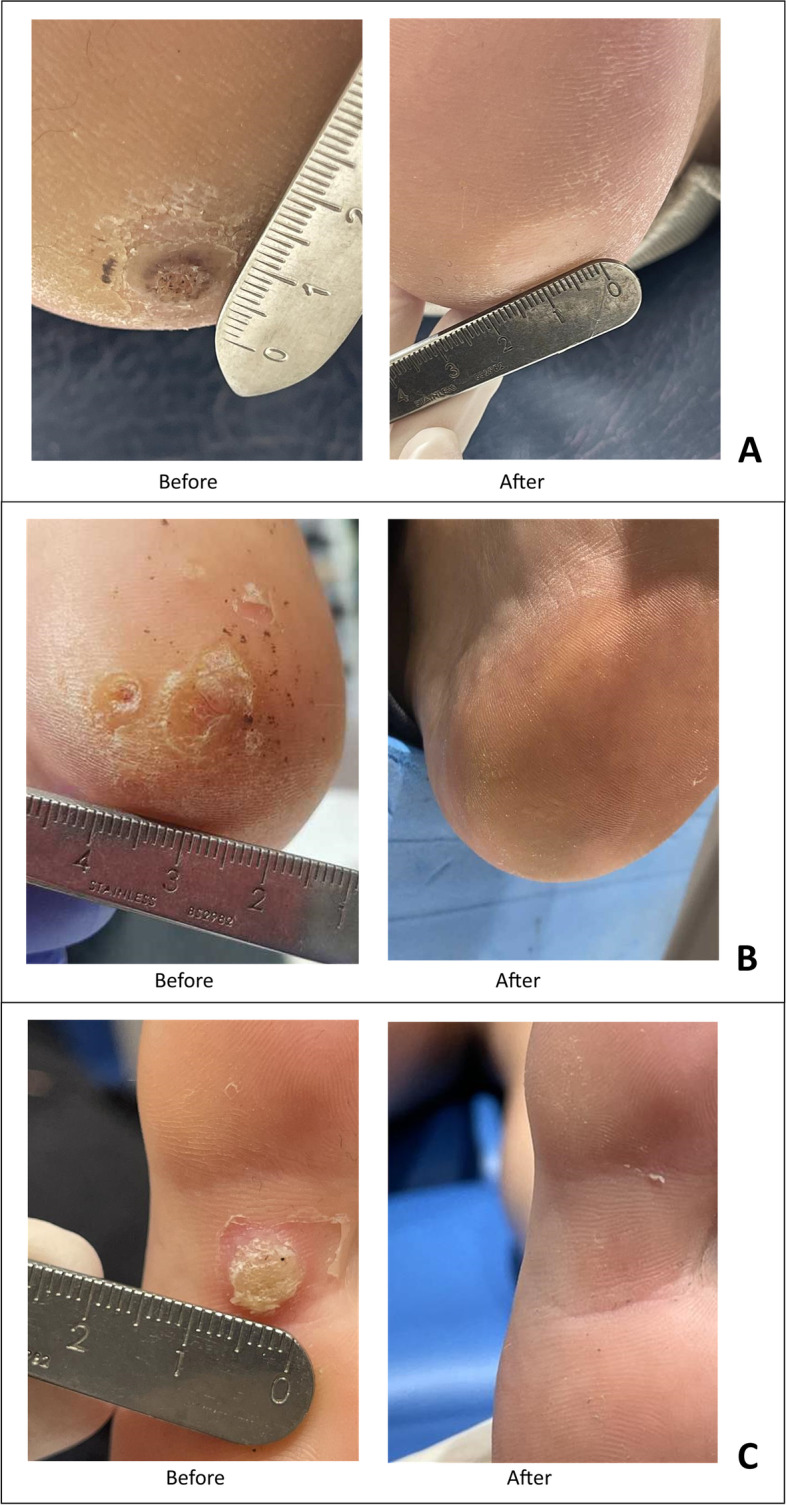
Before and after microwave therapy. **A** right plantar heel; (**B**) left plantar heel; (**C**) left plantar hallux

In 32 patients full lesion resolution was achieved and in 10 patients lesion resolution was not achieved. Three patients with multiple plantar warts had both resolved and unresolved lesions. Of the total 150 total plantar warts treated with microwave therapy, 125 (83.3%) warts resolved and 25 (17%) warts did not resolve. Figure 2 presents examples of plantar lesions pre- and post-treatment. In the generalised linear model only decreasing age was a significant predictor of plantar wart resolution (odds ratio (95% confidence interval), 0.84 (0.71, 1.00), *P* = 0.046). The model explained 99.7% of the variance in plantar wart resolution (conditional R^2^).

## Discussion

This retrospective study has shown that three to four sessions of controlled heating of keratinised skin with microwave therapy resolves the majority of longstanding plantar warts. The complete resolution rate of 83.3% is consistent with the resolution rate of 75.9% reported in a previous pilot study involving 32 patients with 54 recalcitrant plantar warts who were treated with microwave therapy [18]. These rates are substantially higher than the resolution rates for commonly used cryotherapy (45.6%) and salicylic acid (13.6%) and intralesional immunotherapy (68.1%) [22]. The analysis has also shown that lesion resolution is associated with a younger age which is consistent with existing research where both natural resolution and therapeutic cure rates for plantar warts across a number of different therapies are higher in younger populations [23, 24].

Compared with other commonly used therapies, which can result in pain, bleeding, secondary infection and ulceration [25], participants receiving microwave therapy in this study required no post-treatment recovery period. Although participants reported considerable pain during the application of microwave therapy, on departure from the appointment their pain levels had reduced to baseline, highlighting the short-lived nature of pain associated with this treatment. Participant-reported pain during treatment was significantly higher for unresolved lesions compared to those that resolved, which was likely due to the greater average joules used per treatment session for these more recalcitrant lesions.

The results of this study should be considered in light of some limitations. Firstly, the retrospective nature of the study design and the exclusion of data from patients not responding to the recruitment invitation may have introduced selection bias (i.e., patients who felt more strongly about the success of the treatment they received may have been more willing to participate) [26]. There was also missing data related to lesion duration and pain scales for some participants and there was no documentation of other factors which may have affected lesion resolution, including type of wart and HPV genotype. The patient sample size was also small, however most patients had multiple lesions which contributed to reducing type II errors and increasing power in the analysis. To confirm the efficacy of microwave therapy for the treatment of plantar warts a larger randomised trial is warranted.

In conclusion, this retrospective study of 45 participants has shown that plantar warts may be resolved with two to three sessions of microwave therapy, which may be more successful in younger populations.

## Data Availability

The datasets used and/or analysed during the current study are available from the corresponding author on reasonable request.
